# Dr. Joseph Nelson

**Published:** 1910-09-10

**Authors:** 


Dr. Joseph Nelson, who died at Belfast last week, served under Garibaldi as a member of the British brigade, and took part in several engagements, subsequently going to India, where he lived for some years. He was a native of County Down, and during the last quarter of a century occupied a leading position in the medical profession in Ulster, specialising in throat and ear surgery. He was a member of the Council of the Ophthalmic Society of the United Kingdom, ex-President of the Ulster Medical Society, and surgeon to Belfast Royal Hospital and to Belfast Hospital for Children.

